# Supplementary material for: ERα36–GPER1 Collaboration Inhibits TLR4/NFκB-Induced Pro-Inflammatory Activity in Breast Cancer Cells
Source: Int J Mol Sci. 2021 Jul 16;22(14):7603. doi: 10.3390/ijms22147603 (PMC8303269; doi:10.3390/ijms22147603)
Supplement: Supplementary file 1 [file ijms-22-07603-s001.zip › ijms-1280880-supplementary.pdf]

International Journal of Molecular Sciences

MDPI

Supplemental Tables

Table S1. Primers used for the PCR studies.

Gene	Forward Primer	Reverse Primer
ERα66	AATTCAGATAATCGACGCCAG	TTTCAACATTCTCCCTCCTC
ERα36	CCAAGAATGTTCAACCACAACCT	GCACGGTTCATTAACATCTTTCTG
GPER1	TGGTGGTGAACATCAGCTTC	TGAGCTTGTCCCTGAAGGTC
CXCL12	GGGCTCCTACTGTAAGGGTT	TTGACCCGAAGCTAAAGTGG
CXCR4	GGTGCTGAAATCAACCCAC	CGTGGAACGTTTTTCCTGTT
cMYC	CACCGAGTCGTAGTCGAGGT	TTTCGGGTAGTGGAAAACCA
TNFα	AGATGATCTGACTGCCTGGG	CTGCTGCACTTTGGAGTGAT
IL-6	GTCAGGGGTGGTTATTGCAT	AGTGAGGAACAAGCCAGAGC
PD-L1	TATGGTGGTGCCGACTACAA	TGGCTCCCAGAATTACCAAG
Cyclophylin A	ATGGTCAACCCCACCGTGT	TTCTGCTGTCTTTGGAACTTTGTC

Table S2. Binding simulation studies of ERα and ERα36 with GPER1 and NFκB. X denotes either a non-measurable binding or a measurable yet non-expected binding. NR = Non-relevant information, either because the molecule did not participate in the complex or because of a non-identified molecular interaction.

MONOMERS					
No	Complex 1 (ligand)	Complex 2 (ligand)	ΔG (Kcal/mol)	NLS ERa36	NLS NF-kB
1	GPER1	ERa36	−565.19	BLOCKED	NR
2	GPER1(Estradiol)	ERa36	X	NR	NR
3	GPER1(Estradiol)	ERa36(Estradiol)	−238.74 (X)	BLOCKED	NR
4	GPER1	ERa36(Estradiol)	−296.07 (X)	FREE	NR
5	GPER1	G_{ai}GDP	X	NR	NR
6	GPER1(Estradiol)	G_{ai}GDP	−689.54	NR	NR
7	GPER1(Estradiol)-G_{ai}GDP	ERa36	X	NR	NR
8	GPER1(Estradiol)-G_{ai}GDP	ERa36(Estradiol)	−218.27 (X)	FREE	
9	GPER1	G_sGDP	X	NR	NR
10	GPER1(Estradiol)	G_sGDP	X	NR	NR
11	ERa36	NFkB	X	NR	NR
12	ERa36(Estradiol)	NFkB	−312.60 (X)	FREE	FREE
13	ERa36(Estradiol)-NFkB	GPER1	−676.75	FREE	FREE
14	ERa36(Estradiol)-NFkB	GPER1(Estradiol)	−815.93	FREE	BLOCKED
15	GPER1	NFkB	−958.32	NR	FREE
16	GPER1(Estradiol)	NFkB	−1015.87	NR	FREE
17	GPER1-NFkB	ERa36	X	NR	NR
18	GPER1-NFkB	ERa36(Estradiol)	−423.65	FREE	FREE
19	GPER1(Estradiol)-NFkB	ERa36	X	NR	NR
20	GPER1(Estradiol)-NFkB	ERa36(Estradiol)	−450.29	BLOCKED	FREE
21	GPER1-NFkB	ERa66	−797.14	FREE	FREE
22	GPER1-NFkB	ERa66(Estradiol)	−801.41	FREE	FREE
23	GPER1(Estradiol)-NFkB	ERa66	−845.62	FREE	FREE
24	GPER1(Estradiol)-NFkB	ERa66(Estradiol)	−681.13	BLOCKED	FREE
25	GPER1	ERa66	−984.59	BLOCKED	NR

26	GPER1(Estradiol)	ERa66	X	NR	NR
27	GPER1(Estradiol)	ERa66(Estradiol)	X	NR	NR
28	GPER1	ERa66(Estradiol)	X	NR	NR
29	GPER1-ERa36	NFkB	X	NR	NR
30	GPER1-ERa36	NFkB	X	NR	NR
31	GPER1(Estradiol)-G_{ai}GDP	NFkB	−1028.28	NR	FREE
32	GPER1(Estradiol)-G_{ai}GDP-ERa36(Estradiol)	NFkB	−446.71	FREE	FREE
33	GPER1(Estradiol)-G_{ai}GDP-NFkB	ERa36	X	NR	NR
34	GPER1(Estradiol)-G_{ai}GDP-NFkB	ERa36(Estradiol)	−564.81	BLOCKED	FREE
35	GPER1(Estradiol)-G_{ai}GDP	ERa66	X	NR	NR
36	GPER1(Estradiol)-G_{ai}GDP	ERa66(Estradiol)	−918.39	FREE	NR
37	GPER1(Estradiol)-G_{ai}GDP-ERa66(Estradiol)	NFkB	−343.15 (X)	FREE	FREE
38	GPER1(Estradiol)-G_{ai}GDP-NFkB	ERa66	X	NR	NR
39	GPER1(Estradiol)-G_{ai}GDP-NFkB	ERa66(Estradiol)	−569.75	BLOCKED	FREE
		DIMERS			
No	Receptor	Ligand	ΔG (Kcal/mol)	NLS ERa36	NLS NF-kB
1	ERa36(Estradiol)	ERa36(Estradiol)	−609.34	FREE	NR
2	GPER1(Estradiol)-G_{ai}GDP	GPER1(Estradiol)-G_{ai}GDP	−407.97	NR	NR
3	[GPER1(Estradiol)-G_{ai}GDP]$_2$	[ERa36(Estradiol)]$_2$	X	NR	NR
4	GPER1(Estradiol)]$_2$	[ERa36(Estradiol)]$_2$	X	NR	NR
5	[GPER1(Estradiol)]$_2$	NFkB	−508.50	NR	FREE
6	[GPER1(Estradiol)]$_2$-NFkB	NFkB	−1065.20	NR	FREE
7	[GPER1(Estradiol)-NFkB]$_2$	[ERa36(Estradiol)]$_2$	−664.51	FREE	FREE

Supplemental Figures

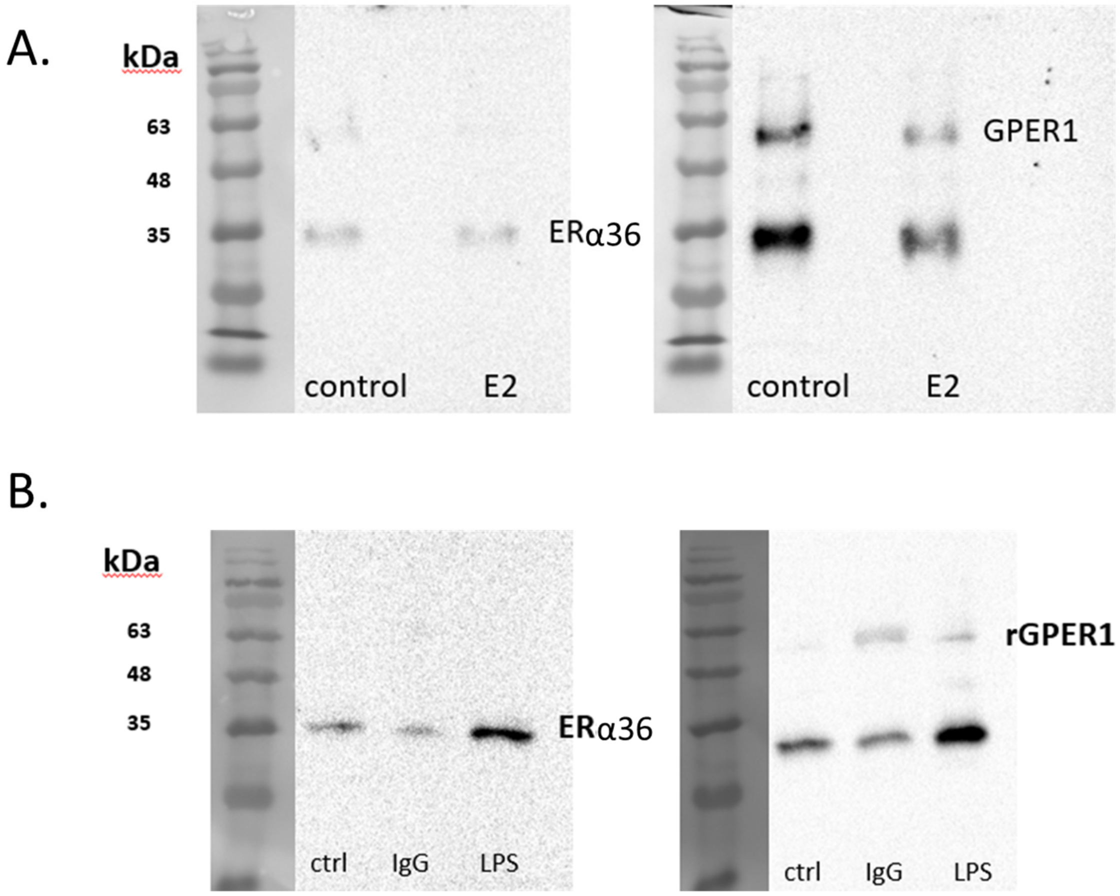

Figure S1. Co-immunoprecipitation studies in SKBR3 cells. A. E2 (10^{-6} M) treatment of SKBR3 cells did not lead to increased precipitation of ERα36 when an anti-GPER1 was used for the initial protein precipitation. B. However, when cells were treated with LPS, an increased amount of ERα36 could be found in the precipitate. All experiments were repeated three times.

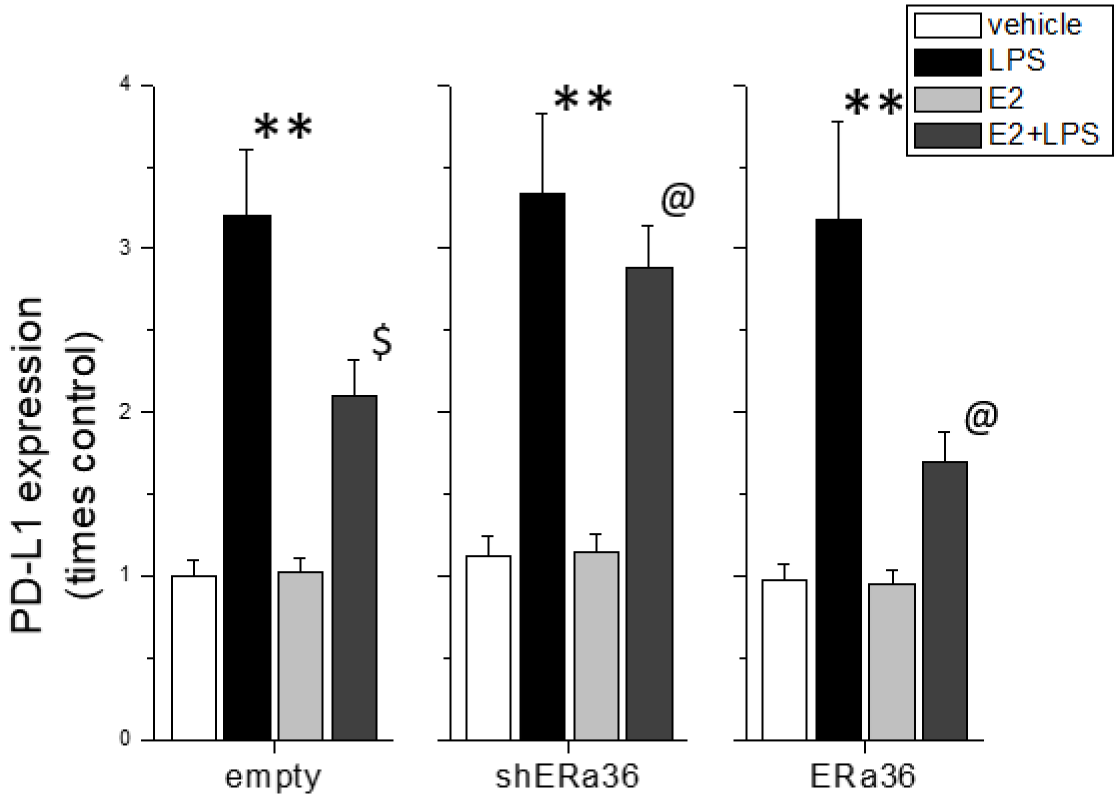

Figure S2. qPCR analysis of PD-L1 expression in SKBR3 cells under conditions of ERα36 knock-down or overexpression. All experiments were repeated three times in triplicates. * $p < 0.05$ and ** $p < 0.01$ vs. vehicle, $ $p < 0.05$ vs. LPS, @ $p < 0.05$ and @@ $p < 0.01$ vs. E2 + LPS in empty, one-way ANOVA with Dunnet's test for multiple comparisons.

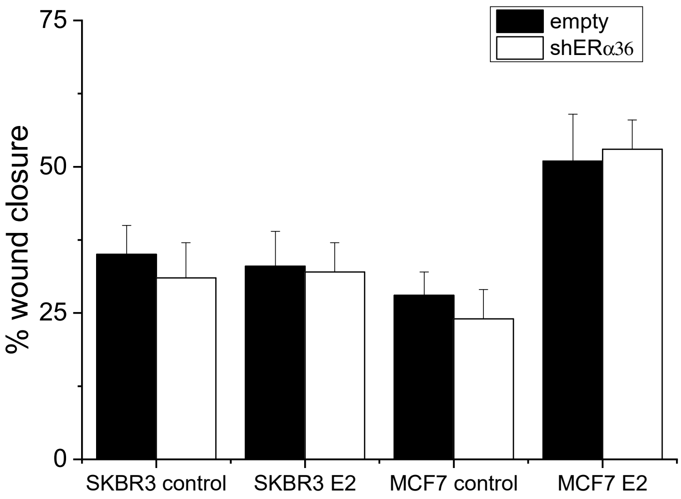

Figure S3. Wound healing assay in SKBR3 and MCF7 cells in the presence and the absence of estrogen. Knock-down of ERα36 expression did not affect the wound healing capacity of the studied cell lines, n = 3.

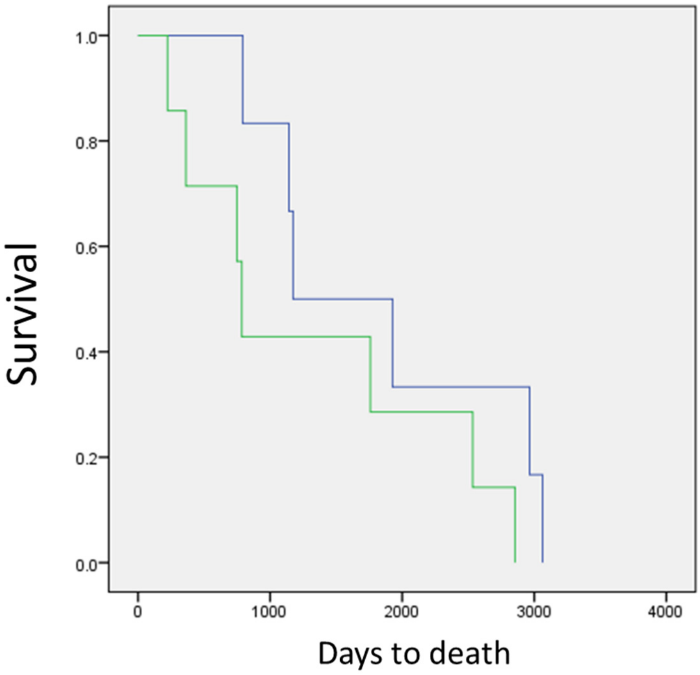

Figure S4. Kaplan–Mayer survival curves of Caucasian breast cancer patients that displayed an expression of ERα36 above (blue) or below (green) the median of the whole TCGA breast cancer cohort. The difference between the two groups was not statistically significant, probably due to the small number of patients with reported cancer-related death in the cohort.
